# Towards a theory of human creativity sustained by embodied collective intelligence

**DOI:** 10.3389/fpsyg.2026.1752280

**Published:** 2026-04-09

**Authors:** Tatsuya Daikoku

**Affiliations:** Graduate School of Information Science and Technology, The University of Tokyo, Tokyo, Japan

**Keywords:** collective intelligence, embodied cognition, empathy, ethics, uncertainty dynamics

## Abstract

Creativity in human societies may be sustained not by isolated minds but by embodied, collective intelligence. Using music as a tractable model system, this paper reviews evidence that creativity depends on the dynamic tuning of uncertainty: moderate, time-varying surprise—often accompanied by interoceptive bodily sensations—can promote exploration, while interpersonal synchrony and social evaluation can stabilize and transmit what is new. Because creativity itself is value-neutral, its social consequences depend on the co-occurrence of ethics, morality, and empathy. This paper argues that ethical norms bound admissible deviation, and empathic perspective-taking—supported by bodily and physiological coupling—acts as a social form of precision weighting that increases (or decreases) the likelihood that novelty is admitted, reshaped, or rejected by a community. From these ingredients emerges field-level intelligence (FLI): a process in which groups preserve diversity, selectively integrate deviations, and maintain “just-right” uncertainty under partially connected social structures. Although music provides a high-resolution measurement sandbox, this paper articulates boundary conditions and testable predictions for other domains (e.g., scientific discovery, design, and everyday collaboration) and for human–AI co-creativity. This paper outlines an empirical agenda: operationalize FLI and social precision weighting, quantify uncertainty dynamics in embodied interaction, and identify network conditions that sustain socially shared creativity without overgeneralizing across domains.

## 1 Introduction

Creativity is a fundamental human capacity that drives societal advancement. It is the ability to generate novel ideas and frameworks and to embed them in forms that can be shared with others. This capacity extends across domains as varied as science and technology, the arts, education, industry, and everyday collaboration (Hennessey and Amabile, 2010). Crucially, creativity is not a transient spark of inspiration; rather, it is a dynamic process that is continuously sustained and renewed across generations and communities. Creativity is not a phenomenon confined to the solitary mind; it emerges, is tested, and ultimately selected through the dynamics of social interaction. Within networks of collaborators and teams, ideas are challenged, refined, and either disseminated or discarded—underscoring that creativity is as relational and collective as it is cognitive (Uzzi and Spiro, 2005; Wuchty et al., 2007). This cumulative renewal of knowledge—often termed the “ratchet effect”—has long been a central theme in cultural evolution research (Dean et al., 2012). Elucidating the mechanisms that sustain creativity is therefore indispensable for understanding the very foundations of sustainable intelligence and the dynamics of cultural development.

A useful contrast for these dynamics is the recent rise of large language models (LLMs). These systems, accessed through natural-language interfaces, have broadened access to knowledge (Brown et al., 2020). Yet their control still relies heavily on *post hoc* alignment, safety filters by humans, and output-level moderation (Ouyang et al., 2022; Gehman et al., 2020; Weidinger et al., 2022). As a result, vulnerabilities remain, including susceptibility to unexpected behaviors and the reproduction of biases inherited from training data (Bender et al., 2021). Moreover, empirical and theoretical work has shown that when models iteratively incorporate their own generated data into retraining, the distributional diversity erodes and both quality and variability decline—a phenomenon termed “model collapse” (Shumailov et al., 2024). In other words, this trend raises the concern that the very source of creativity—the richness of uncertainty and diversity—may be lost. This contrast forces us to confront a fundamental question: why is creativity in human societies far less vulnerable to collapse? Why, instead, is diversity maintained and even amplified across generations? And what mechanisms sustain such a resilient system?

This review addresses these questions from the perspective that creativity is sustained not at the level of the individual, but at the level of the collective. This paper focuses in particular on music as a model system. From the standpoint of predictive processing—namely, the interplay between prediction errors and their precision—music provides an ideal domain in which to examine how novelty is linked to surprise and how new information is assigned “value” or experienced as pleasure (Friston, 2010; Koelsch et al., 2019; Pearce and Wiggins, 2012; Salimpoor et al., 2011). Moreover, decision-making and emotional processes grounded in the norms and violations of musical grammar have been visualized in neural activity (Maess et al., 2001; Koelsch, 2014). The social and collective diversity and universality that arise through music further illuminate a “maintenance system” that enables creativity to be continuously renewed (Mehr et al., 2019). Building on these insights, our aim is to delineate the fundamental mechanisms that sustain the ongoing renewal of creativity in human societies and to propose a theoretical framework that integrates them. Ultimately, this paper extends this perspective beyond music to domains such as scientific research and human–AI collaboration, thereby advancing an interdisciplinary understanding of the fundamental question: how is creativity not only preserved, but actively amplified across generations?

Music is used here as a high-temporal-resolution domain in which predictive models, surprise, and uncertainty can be quantified and experimentally manipulated. Musical creativity is not assumed to be identical to creativity in science, visual arts, or everyday collaboration. Rather, the paper treats music as an existence proof and a measurement sandbox for linking uncertainty dynamics to embodied experience and social coordination, and derives hypotheses that require cross-domain testing. Accordingly, this distinguishes (i) components that plausibly generalize across domains (e.g., “just-right” uncertainty, partial connectivity, and interaction-level selection of deviations) from (ii) components that are likely domain-specific (e.g., ethical norms, institutional gatekeeping, and the cost of errors). This framing motivates a set of operational definitions and falsifiable predictions. Appendix A summarizes the core constructs used throughout the paper along with example operationalizations, and provides a concise glossary for quick reference.

## 2 Sustenance of creativity in human societies

### 2.1 Dynamics of uncertainty emerging from embodied-collective intelligence

The sustained renewal of creativity across generations and communities cannot be explained solely by individual predictive models. Instead, the evidence points toward a fundamentally collective substrate of human predictive processing, continuously shaped through embodied and social interaction. Here, this paper uses “uncertainty” for how unpredictable the next event is (often quantified as entropy) and “surprise” for how much an event violates expectations (prediction error). In predictive processing, “precision” refers to the confidence (roughly the inverse of uncertainty) assigned to prediction errors, which controls how strongly deviations update predictions. This paper uses the term conceptually, and Supplementary lists candidate empirical proxies.

Using music as a predictive model system, this paper has developed an integrative approach that couples predictive processing with behavioral and neuroscientific measurements in humans (Daikoku and Tanaka, 2024). This work has analyzed a large corpus of jazz improvisations spanning the early twentieth to the twenty first century (456 pieces, 78 musicians) with a hierarchical Bayesian statistical learning model (Daikoku et al., 2023; Daikoku, 2023). This analysis revealed that while the acoustic properties of musical signals themselves remain strikingly stable across eras, the temporal dynamics of prediction error (surprise) and entropy (uncertainty) exhibit gradual change, period-specific signatures (Daikoku, 2024). In particular, pitch-related sequences displayed gradual, community-level shifts in their temporal patterns of uncertainty, whereas rhythmic structures—such as fundamental ratios of 1:1 and 1:2 (e.g., a quarter note followed by another quarter note = 1:1, a quarter note followed by a half note = 1:2)—remained relatively invariant across time. The key point is this: historical change is reflected not in the raw acoustic signal, but in the probabilistic structure of sequences—transition probabilities, their uncertainty, and how these fluctuate over time. These findings suggest that the dynamics of deviation and alignment within shared predictive models are what drive creativity as a collective, emergent form of intelligence rather than as an individual cognitive process.

**Figure 1:**
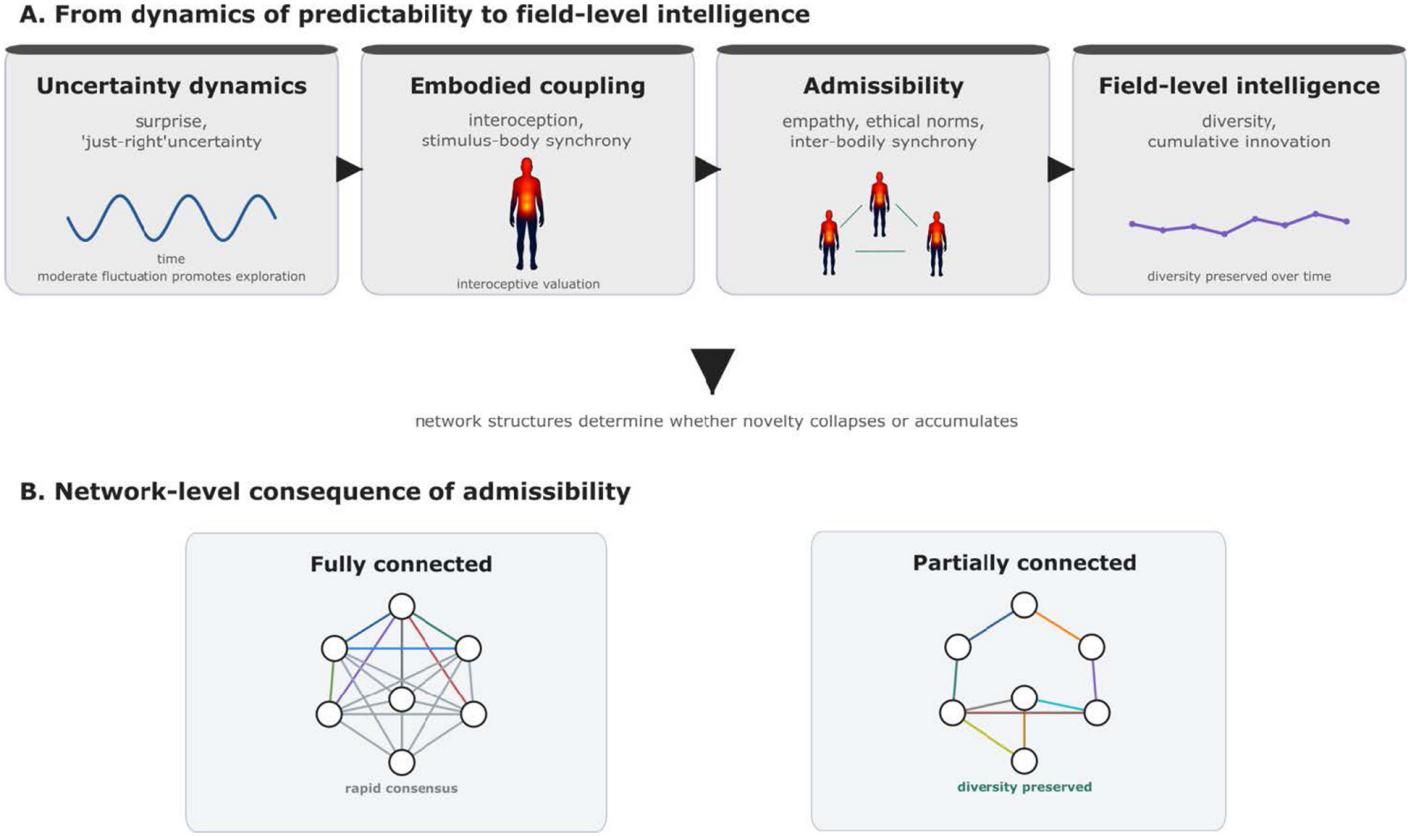
Schematic overview of the proposed framework linking uncertainty dynamics, embodiment, and collective intelligence. **(A)** The upper panel illustrates the hypothesized pathway from creative deviation to field-level intelligence. **(B)** The lower panel illustrates the network-level consequences of admissibility. In fully connected networks, rapid information diffusion tends to produce fast consensus, increasing the risk of premature convergence and reducing diversity. In contrast, partially connected networks allow deviations to persist locally and be explored in parallel, thereby preserving diversity and supporting cumulative innovation over time. Together, the panels illustrate how uncertainty dynamics, embodied valuation, social regulation, and network structure interact to sustain creativity in human collectives.

Complementary studies reinforce this view. Figure 1 schematically summarizes the temporal dynamics of uncertainty in jazz improvisation and chord progressions, together with the proposed bodily-synchrony overlay. In a large-scale experiment with 353 participants, emotional evaluations and bodily maps were collected in response to chord progressions. By constructing a population-level predictive model of chord progression (Daikoku et al., 2024) and manipulating the temporal dynamics of uncertainty and prediction error, this paper demonstrated that chord progressions with higher uncertainty elicited stronger subjective feelings of creativity and heightened arousal (Daikoku and Tanaka, 2024). Importantly, this effect was tightly linked to interoceptive sensations in the cardiac region, whereas the perception of beauty and pleasure was more strongly associated with predictable and less uncertain chord progressions. These results indicate that fluctuations in high uncertainty, grounded in collective predictive models, recruit embodied pathways to promote the feeling of creativity, whereas aesthetic value is stabilized by predictability (for theoretical background, see Critchley et al., 2004; Seth, 2013). Crucially, these findings are consistent with broader cross-cultural evidence on the embodied grounding of affect and music. Volynets et al. (2020) demonstrated that bodily maps of basic emotions are culturally universal, suggesting that interoceptive patterns provide a shared biological template for linking internal bodily states with affective experiences. Extending this framework, Putkinen et al. (2024) revealed that bodily maps of musical sensations likewise display systematic and cross-culturally consistent patterns. Together, these results highlight that the coupling between predictive dynamics and embodied sensations is not culturally idiosyncratic but reflects a universal mechanism through which human societies anchor and transmit affective and creative experiences (Daikoku and Tanaka, 2026). Taken together, these converging findings underscore a key principle: creativity is not simply sustained by novelty *per se*, but by the temporal dynamics of uncertainty embedded in collective predictive frameworks, which engage the body and its interoceptive signals as mediators of creative experience. This perspective aligns with recent affective neuroscience and music cognition studies showing that both surprise and uncertainty fluctuations shape emotional intensity and social bonding in music (e.g., Vuust et al., 2022b). Moreover, the cross-cultural universality of bodily maps of both emotions and musical sensations may suggest that embodied pathways form a robust and species-wide foundation for the resilience and renewal of creativity in human societies.

These observations reinforce the view that value judgments—including those underlying creativity—do not reside within the isolated individual but emerge in social and collective contexts of resonance. Neural evidence has shown that coupling of neural activity between speakers and listeners, or performers and audiences, is a critical determinant of mutual understanding and joint action in language, music, and gesture (Stephens et al., 2010; Hasson et al., 2012). Likewise, physiological synchronization during collective rituals may be linked to the strengthening of social bonds (Konvalinka et al., 2011; Hove and Risen, 2009; Mogan et al., 2017), while synchronized movement more broadly promotes cooperative behavior (Wiltermuth and Heath, 2009). Together, such findings highlight how “collective intelligence” can emerge from the interactions of diverse individuals (Woolley et al., 2010). Diversity itself plays a decisive role: heterogeneous groups consistently outperform homogeneous ones (Hong and Page, 2004), and success in cultural markets often follows unpredictable and unequal trajectories driven by social influence (Salganik et al., 2006). These strands of evidence converge on a central principle: the value of creativity is distributed across a collective/social “field,” shaped through the alignment and deviation of mutual predictions.

The computational basis of such dynamics can be situated within predictive processing frameworks, which emphasize the representation and weighting of uncertainty in Bayesian inference (Knill and Pouget, 2004; Körding and Wolpert, 2004). Humans appear to prefer an intermediate rate of information—neither too little nor too much—a phenomenon known as the “Goldilocks effect,” observed in infants' allocation of visual and auditory attention (Kidd et al., 2012). This preference for “just-right” uncertainty resonates with musical groove, where moderate deviations in prediction sustain engagement and drive affective responses (Vuust et al., 2022a). It also suggests a precision-tuning mechanism that regulates the optimal balance of pleasure, arousal, and immersion that defines creative experience. At the societal level, the dynamics of maintaining uncertainty without excess or depletion resemble probabilistic search algorithms. For instance, the Metropolis–Hastings algorithm accepts even rare deviations with a finite probability, thereby preventing premature convergence and preserving diversity (Metropolis et al., 1953; Hastings, 1970). Experimental studies of cultural evolution further support this analogy: partially connected networks, which avoid overly dense synchronization, foster the accumulation of diverse and complex solutions (Derex and Boyd, 2016). Figure 1 contrasts fully connected and partially connected networks, highlighting why moderated connectivity can preserve diversity while avoiding premature convergence.

To sharpen the analogy with probabilistic search, it can be mapped (i) a creative deviation to a proposed candidate state, (ii) the field's evaluative landscape to a (time-varying) target distribution over admissible states, and (iii) social uptake to an acceptance step whose probability increases with expected value but decreases with expected social and ethical cost. In this view, a tolerance parameter captures how permissive a community is toward exploratory deviation. This mapping is heuristic rather than literal: real creative fields are non-stationary, multi-criteria, and institutionally constrained. Nevertheless, the mapping makes a concrete point—finite acceptance of imperfect deviations can prevent premature convergence and help sustain long-run diversity.

Taken together, these findings suggest that human creativity is sustained not by optimizing individual internal models, but by collective processes of acceptance and selection mediated through bodily synchronization, emotional resonance, and reciprocal prediction. Accordingly, intelligence and creativity are better understood at the level of interaction patterns than at the level of isolated individuals alone. These structures flexibly regulate the temporal profile of surprise and uncertainty, allowing norms and deviations to coexist in dynamic tension. It may be precisely this capacity for continual adjustment that safeguards prediction uncertainty as the wellspring of creativity.

### 2.2 Co-occurrence of ethics, morality, and empathy

Having outlined how uncertainty dynamics are embodied and socially shared, this paper next considers how novelty is regulated by social norms and social understanding. The social and collective dynamics that sustain creativity do not terminate within individual brains. This paper proposes that three processes intertwine within collective dynamics, forming what can be viewed as an “intelligence of fields”: (i) innovative ideas that underlie uncertainty; (ii) ethical/moral constraints that stabilize predictability by delimiting what is acceptable; and (iii) empathy and perspective taking, which bridge the two by rendering innovation intelligible and admissible to others.

Empirical evidence supports the key components of this framework. Individuals with stronger creative problem-solving skills show superior ethical decision-making (*N* = 258), and creative reasoning can unlock moral insights that reconcile value conflicts—particularly when actors generate multiple “What could I do?” options (Mumford et al., 2010; Zhang et al., 2018). On the other hand, creativity has a dark side. Creativity can facilitate the self-justification of unethical conduct (e.g., creatively finding loopholes) and is associated with increases in dishonest behavior (Gino and Ariely, 2012). Furthermore, malevolent creativity—novel and effective ideas serving harmful purposes—has been theorized and functionally modeled in domains such as terrorism and crime (Cropley et al., 2008). These findings imply that creativity *per se* is value-neutral; the field conditions that align innovations with social norms—and thereby shape the probability of acceptance—determine whether novelty crystallizes as ethical innovation or deviates into harmful ingenuity.

Within this regulatory framework, empathy and perspective taking may function not merely as correlates of social behavior but as mechanisms through which a social field assigns precision to prediction errors arising from others' actions. In predictive processing terms, when another agent introduces a deviation from shared expectations, this generates a social prediction error. Whether such a deviation updates a shared model depends on the precision assigned to it—that is, the inferred reliability of the signal and the anticipated consequences of adopting it. This paper proposes that empathic cues—such as inferred benevolent intent, shared affect, or physiological synchrony—can increase the perceived reliability of the deviating agent and reduce expectations of harm. In doing so, they effectively increase the precision assigned to the deviation, thereby raising the probability that it will be explored and integrated rather than rejected. Conversely, cues signaling malicious intent or norm violation reduce the assigned precision, suppressing learning from the deviation even when it is highly surprising. The causal claim advanced here is modest: empathic and norm-related cues are hypothesized to alter the precision assigned to deviations, which in turn changes whether they are explored, adopted, or rejected. This mechanism is not uniformly prosocial; in domains such as advertising or political messaging, affective attunement and perspective taking can also amplify ethically ambiguous or manipulative deviations, underscoring that empathy-like alignment must be constrained by explicit norms.

This interpretation is consistent with converging evidence from affective neuroscience linking empathy to interoceptive processes. Neural activity in regions associated with interoceptive awareness—particularly the anterior insula—has been shown to contribute to empathic experience (Critchley et al., 2004). Similarly, heartbeat-evoked potentials (HEP) have been implicated in the neural representation of others' affective states (Fukushima et al., 2011). Interoception—the perception and interpretation of internal bodily states—provides a critical substrate through which prediction errors are coupled to affective value. The framework of interoceptive inference further suggests that emotional experience emerges from predictive regulation of bodily signals, whereby prediction errors about internal states shape subjective feeling and behavioral responses (Seth, 2013). In this sense, empathy may be understood as a process through which representations of others become integrated with predictive models of bodily states, allowing deviations introduced by others to acquire affective and motivational significance.

Beyond its neural grounding, empathy operates socially by shaping how novel deviations are interpreted and evaluated within collective contexts. Perspective taking—the capacity to adopt another person's standpoint and infer their intentions—plays a particularly important role in this process. In negotiation research, perspective taking facilitates the discovery of hidden agreements and enables the creation of joint value (Galinsky et al., 2008), while improving outcomes in mixed-motive situations (Gilin et al., 2013). It also promotes intergroup contact and increases cohesion across social boundaries (Wang et al., 2014). From this perspective, perspective taking may transform exploratory deviations into forms that are intelligible to others. When combined with interoceptive bodily signals and interpersonal synchrony, this process allows creative mismatches to be socially interpreted rather than dismissed. In doing so, empathy and perspective taking help regulate the balance between exploratory creativity and ethical acceptability, enabling social systems to incorporate novelty without collapsing into either rigid conformity or uncontrolled deviation.

These mechanisms are particularly vivid when performing music, where interactions among creative behavior, morality/ethics, and empathy prevent both collapse from excessive novelty and stagnation from rigid adherence to norms. In a jazz session, the groove established by the rhythm section scaffolds the soloist's bold and novel insertions; the ensemble and audience momentarily hold their breath as a bodily response to surprise, but then stabilize interpretation and predictability by catching the phrase harmonically and metrically. In these processes, performers, listeners, and co-listeners alike become physically coupled to the music, their bodies moving spontaneously (Janata et al., 2012). Such bodily entrainment naturally gives rise to interpersonal synchrony, which in turn enhances affiliation (Hove and Risen, 2009). Building on this foundation, evidence shows that joint music-making promotes prosocial behavior (Kirschner and Tomasello, 2010), while group singing and synchronous dance strengthen social bonding (Tarr et al., 2014; Weinstein et al., 2016). From lullabies and choral singing to the dance floor, this loop re-emerges across contexts, sustaining a dynamic balance between novelty and stability—likely grounded in musical moral–ethical sensibilities and collectively negotiated through embodied empathy and perspective taking.

Meta-analyses indicate that interpersonal synchrony in musical activities (choral singing, ensemble performance, dance) increases cooperation, altruism, and cohesion (Mogan et al., 2017; Rennung and Göritz, 2016). Synchrony heightens self–other overlap and—sometimes via endogenous opioids—boosts trust and cooperation (Lang et al., 2017). In this light, the ethics of musical practice can be viewed as the coordination rules that sustain “*creative exploration within a shared temporal action space*” by satisfying: (1) care (protecting others' agency and welfare), (2) fairness (equitable distribution of participation and resources—time, loudness, solos, credit), and (3) authority/norm-respect (adherence to agreed forms, rituals, and copyrights) as foundations in moral foundations theory (Graham et al., 2018). These may be scaffolded by bodily synchrony and perspective taking. The result is the maintenance of socially optimized uncertainty, steering between the twin failure modes of over-stability (rigidity) and over-uncertainty (chaos). The next section integrates these ingredients—uncertainty dynamics, embodied coupling, and normative/empathic regulation—into a field-level account of how collective intelligence can sustain creativity over time.

## 3 Collective intelligence emerging from uncertainty dynamics mediated by creativity, ethics, and empathy

The foregoing arguments indicate that the sustainment of creativity cannot be explained from an individual's internal model. Rather, it depends on collective interaction grounded in embodied sensibility. In music, for example, the timing of prediction and deviation is socially shared; the resulting mismatch and surprise are experienced as a communicable sense of creativity through bodily synchrony and resonance among performers and listeners. Crucially, what supports creativity is not novelty *per se*, but the temporal dynamics of predictive uncertainty. Novelty functions as a probabilistic fluctuation that is continuously modulated by the group and distributed through bodily experience, rather than as a single, isolated jolt.

Creativity is likewise inseparable from ethics/moral/insight and empathy. While it can generate moral insight, it also carries the risk of norm violations and self-justification. This ambivalence is governed by social regulatory mechanisms—bodily synchrony, empathy, and perspective taking. Whether a creative deviation is admitted or rejected by a community is decided within a social field through embodied empathic understanding and its alignment with ethical norms. By “collective,” this paper therefore does not mean a mere aggregation of parallel individuals. The term designates an emergent, field-level intelligence (FLI) that arises from the dynamics of bodily coordination and misalignment. Operationally, FLI refers to a group's capacity to preserve diversity, selectively admit useful deviations, and sustain cumulative innovation over time; candidate observables therefore include diversity retention, acceptance rates of borderline deviations, and longitudinal innovation uptake under different network structures. This intelligence of the field continuously fine-tunes between stabilizing norms and exploratory deviations, avoiding both rigidity and chaos. Through this ongoing micro-regulation, creativity should be continually renewed and transmitted across generations and cultures.

Jazz improvisation exemplifies this process. When a soloist launches a bold phrase, listeners' bodies register a brief rise in uncertainty (e.g., faster heartbeat). The ensemble then absorbs the deviation by adjusting harmony and meter, turning it into shared, intelligible invention. Rather than rejecting deviation and novelty, the group incorporates momentary misfit into the field, allowing musical creativity to be updated while retaining ethical and empathic meaning. A similar logic operates in choral singing and joint rhythmic activity. Synchrony of voices and movement creates strong collective cohesion. Individual predictions are calibrated with respect to others, and small deviations are folded back into the overall harmony. Here, normative constraints (pitch, rhythm and musical grammar) remain salient, yet micro-deviations are tolerated as the signature of living music, often enriching creative vitality. Bodily synchrony thus sustains ethical order while keeping creativity alive.

The same dynamics appear in human–AI collaboration, particularly with generative systems. Model outputs often contain unexpected deviations or noise. Through human interpretation and empathic uptake, these fragments can be converted into creative ideas; without such alignment to empathic and ethical constraints, they are dismissed as harmful or meaningless. Hence, even in human–AI settings, embodiment- and empathy-based social regulation is pivotal, opening the possibility of a genuinely co-creative field intelligence spanning humans and machines.

Taken together, uncertainty dynamics mediated by creativity, ethics, and empathy are observable in musical practice. What persists is not a simple sum of individual knowledge but a collective intelligence of the field that rises through the interplay of misalignment and coordination. This structure plausibly underwrites the robustness and sustainability of human creativity. This paper now turns to a broader discussion of implications (including human–AI co-creativity), limitations, and concrete steps for operationalizing and testing the framework.

## 4 Discussion

Intelligence in human societies is rarely the mere sum of individual abilities. Rather, it frequently arises from the space of interaction—the dynamic field of exchanges among people. This emergent capacity is often termed collective intelligence, and group performance on complex tasks is frequently explained less by the distribution of individual IQs than by the quality of coordination within the group (Woolley et al., 2010). Beyond a simple aggregation of minds, intelligence is distributed across bodies, temporal synchrony, and environments, thereby functioning as a field-level phenomenon that extends cognition into the world (Clark and Chalmers, 1998). This paper refers to this distributed, interaction-born capacity as field-level (collective) intelligence. This framework does not deny individual variation in creative ability; rather, it proposes that collective outcomes arise from the interaction between individual-level variability and network-level coordination.

Although music is a privileged measurement domain for timing, prediction, and synchrony, the proposed components—uncertainty regulation, interaction-level selection of deviations, and norm-bounded admissibility—are not inherently musical. In scientific creativity, for instance, novelty is filtered through peer review, replication, and ethical oversight; in everyday collaboration, novelty is filtered through trust, role expectations, and local norms. A scientific example is CRISPR (Jinek et al., 2012): the core methodological innovation diffused rapidly, while ethically sensitive applications remained subject to explicit normative debate and governance. These fields differ in the cost of errors and in the kinds of norm violations that matter, which implies that the same magnitude of “surprise” can be welcomed in one domain and rejected in another. Therefore, generalization requires specifying which variables carry across domains (e.g., uncertainty dynamics and network connectivity) and which variables are domain-specific (e.g., the normative cost function and institutional gatekeeping). This distinction also clarifies how the framework can be used productively: music provides measurable proxies, while cross-domain tests establish external validity. Useful non-musical test beds include scientific collaboration, collaborative design, and human–AI co-creation, where novelty is evaluated under explicit institutional or user-level constraints. Figure 1 schematizes the proposed framework, linking uncertainty dynamics, embodied coupling, ethical filtering, and partial connectivity. A non-musical illustration is CRISPR, where novelty was rapidly adopted as a methodological advance while ethically sensitive applications remained subject to strong normative filtering.

Contemporary neuroscience frames the brain as a predictive organ that minimizes prediction error (Friston, 2010; Knill and Pouget, 2004). Crucially, this predictive machinery is not confined within individuals: during communication, neural activity couples between speakers and listeners, supporting shared understanding and joint action (Stephens et al., 2010; Hasson et al., 2012). In this sense, prediction and error correction are not only intra-cranial processes but also shared across the social field that links brains together. Music is a particularly tractable model system for examining such collective dynamics and FLI. Musical experience hinges on probabilistic “grammars”: listeners form expectations about upcoming notes and chords, and affective responses arise from the balance of confirmation and violation (Koelsch, 2014; Pearce and Wiggins, 2012). A moderate degree of fluctuation of predictability—neither too little nor too much—maximizes pleasure and movement tendencies (Vuust et al., 2022a; Stupacher et al., 2022). Harmony violations recruit regions that also support syntactic processing in language, including Broca's area (Maess et al., 2001). Put simply, when listening to Western tonal music, many listeners strongly expect a tonic after a dominant. Resolution produces feelings of beauty and relief; deliberate detours produce surprise and tension which, when subsequently integrated back into the prevailing grammar, are often shared and appraised as creative (Daikoku et al., 2024). Thus, musical affect emerges from time-varying play between prediction and deviation that is scaffolded by culturally shared grammatical regularities. Music thereby functions as a collective prediction device—a window onto the mechanisms of FLI.

The substrate of this field is bodily. Interoceptive pathways—particularly those involving the anterior insula—support predictions about cardiac and visceral states and the processing of interoceptive prediction errors, shaping emotion and the sense of self (Critchley et al., 2004; Seth, 2013). People also share broadly similar bodily maps of emotion—for example, heat or pressure in the chest for anger or sadness—suggesting a common bodily “lexicon” for affect (Nummenmaa et al., 2014; Daikoku et al., 2026). These embodied templates provide a shared substrate through which affective experiences can be communicated and socially stabilized, thereby supporting collective creativity. Recent work extends these insights directly to music. Cross-cultural mapping shows that bodily sensations evoked by music exhibit systematic topographies that align across Western and East Asian participants—for example, danceable, joyful tracks map more strongly to the limbs, while gentle, sad tracks map to the chest (Putkinen et al., 2024). This pattern generalizes Nummenmaa's bodily maps of basic emotions to the musical domain, suggesting that the interaction among prediction of external phenomenon, bodily sensation, and emotion is, in broad outline, culturally shareable (Putkinen et al., 2024). Such a shared bodily communication makes it easier for performers and audiences to achieve understanding and synchrony, providing a scaffold for collective intelligence. Complementing this, body-map studies focused on the felt sense of creativity show that the experience of creativity tracks the temporal dynamics of uncertainty and surprise in chord progressions. Higher entropy and surprise increase ratings of “felt creativity” and are accompanied by stronger sensations in the cardiac region; by contrast, beauty and pleasantness peak under more predictable, low-uncertainty progressions (Daikoku and Tanaka, 2026). Individuals high in interoceptive sensibility report stronger creative feelings and clearer bodily sensations (Daikoku and Tanaka, 2024). Hence, creative experience is likely mediated not only by informational novelty but also by interoceptive prediction-error processing and its conscious access. In musical settings, momentary mismatches can thus be absorbed via the body into empathic synchrony, and thereby transformed into socially shared judgments of “creative and interesting.”

Taken together, (1) cross-culturally shared emotion–body mappings (Putkinen et al., 2024) and (2) the coupling between uncertainty dynamics and interoception in the experience of creativity (Daikoku and Tanaka, 2024) jointly explain why music readily functions as a collective prediction device. On a widely legible bodily substrate, moderate fluctuations in predictability ignite a sense of creativity that is then distributed through bodily synchrony and empathy into the shared field. This circulation—the rise of creative feeling on a shared bodily code, its uptake through synchrony, and its stabilization in collective appraisal—captures a core mechanism of FLI.

A distinctive strength of collective intelligence is its ability to maintain diversity while keeping uncertainty at a just-right level. Heterogeneous groups tend to outperform homogeneous high-ability groups (Hong and Page, 2004). In cultural evolution experiments, partially connected networks (in which not everyone is tightly linked to everyone else, slowing premature consensus and preserving diversity)—avoiding over-dense coupling—accumulate more diverse and complex solutions than fully connected ones (Derex and Boyd, 2016). Algorithmically, such dynamics echo search schemes that accept rare deviations with finite probability (e.g., Metropolis-Hastings), thereby preventing premature convergence (Metropolis et al., 1953; Hastings, 1970). The Collective Predictive Coding (CPC) framework (Taniguchi et al., 2016; Taniguchi, 2025; Taniguchi et al., 2025) seeks to formalize these ideas computationally. CPC emphasizes that predictive models are not only individual constructs but are also generated and calibrated within the social field, which regulates whether deviations are admissible as shareable innovations. This view aligns with theories of symbol emergence, in which meanings and norms arise not top-down but through interaction, progressively stabilizing within the field (Taniguchi et al., 2016).

To reduce conceptual density and increase falsifiability, this paper proposes several concrete predictions that can be tested across domains: (1) Just-right uncertainty: Perceived creativity/interest and long-run adoption will show an inverted-U relationship with uncertainty (entropy rate or prediction-error variance), both in music and in non-musical sequential tasks, (2) Social precision weighting: Empathy, trust, and physiological synchrony will increase the acceptance probability of borderline deviations when expected harm is low, but will have little or negative effect when ethical risk is high, (3) Partial connectivity: Partially connected communication networks will preserve higher diversity and support cumulative innovation over time compared with fully connected networks.

These “musical collective intelligence” principles may sketch design cues for future AI. Current AI systems—especially LLMs—lack humanlike bodies and therefore struggle to ground symbols in perception and action. This long-standing symbol grounding problem highlights a structural gap between current AI and embodied human cognition (Harnad, 1990). Decades of work in grounded and extended cognition show that human understanding is deeply embedded in body and environment, implying qualitative differences between human learning and prevailing AI pipelines (Barsalou, 2008). The gap is stark for affect: inferring emotion from surface labels or facial/vocal cues alone is insufficient, casting doubt on whether present AI understands emotion in a human-relevant sense. Although affect detection using physiological signals has progressed (Calvo and D'Mello, 2010; Koelstra et al., 2011), AI systems themselves lack interoceptive generative models that predict and correct internal bodily states—the very machinery that supports affect and self in humans (Critchley et al., 2004; Seth, 2013). Consequently, they may poorly be coupled to the bodily synchrony and empathy that scaffold FLI. Relatedly, recent creativity scholarship argues that generative systems at most exhibit “artificial creativity”—outputs that can appear original and effective while lacking the authenticity and intentional agency characteristic of human creative processes (Runco, 2023). Complementing this view, Lockhart (2025) emphasizes that human creativity is grounded in lived, embodied experience and moral agency, which current machine generation does not instantiate.

Music suggests how to close part of this gap. In musical practice, deviations are not discarded as noise but admitted as creativity when they are absorbed through bodily sensation and empathic uptake. Translating this into AI design requires going beyond rule-based governance of morality and ethics to a sensory–affective alignment layer in which systems optionally respond to bio-backed signals—heart rate, respiration, or HRV—under strict privacy-preserving protocols (Critchley et al., 2004; Thayer et al., 2012). A feasible near-term version would not attempt to endow AI with genuine interoception; rather, it would use opt-in human physiological signals as contextual inputs in tightly bounded co-creative settings, building on precedents from affect-aware systems that already estimate human state from physiological data. This should be read as a testable interface proposal, not as a claim that current AI can reproduce human affective embodiment. To avoid model collapse caused by training on self-generated data (Shumailov et al., 2024), novelty should be filtered through multi-agent, heterogeneous reception rather than a single average preference, leveraging diversity's first-order benefits and partially connected evaluation networks that preserve exploration (Derex and Boyd, 2016). In parallel, interactions among humans and AIs can incorporate synchrony-like signals—overlap in attentional allocation, coordinated response latencies, and measures of interpretive agreement—coupled to ethical metrics so that the admissibility of deviations is tuned by the state of the shared field. A practical illustration is the use of generative design-assistance tools in commercial workflows, where AI-generated variations are surfaced for human selection and revision under user control and responsible-AI guardrails rather than being treated as autonomously valid outputs. A further lesson is to regulate “just-right” uncertainty. Inspired by the Goldilocks effect, systems should maintain moderate predictive variability—neither boring nor chaotic—by adaptively controlling sampling temperature, search breadth, and presentation order (Kidd et al., 2012). Conceptually, these ingredients align with Collective Predictive Coding: meanings and norms emerge through interaction (Taniguchi et al., 2016, 2025), and the social field dynamically assigns precision to deviations—accepting, reshaping, or rejecting them in context. Operationalizing these mechanisms would begin to instantiate FLI in AI, opening a path toward systems that cohere with human social dynamics rather than merely optimizing in isolation.

Several limitations qualify the scope of this review. First, the strongest empirical demonstrations reviewed here come from music-cognition studies, so the present framework should be read as a music-grounded proposal rather than a cross-domain law. The extent to which the proposed mechanisms generalize to other coordination-intensive domains—scientific collaboration, education, or collective decision-making—remains uncertain, and the rhythmic invariances discussed here are best treated as domain-specific observations until analogous tests are conducted outside music. Even where cross-cultural similarities in bodily maps for music are observed, cultural ecologies may still shape norms for “admissible deviation” and sanctioning in ways this paper has not fully captured. Second, constructs such as FLI and admissibility of deviation risk remaining theoretical unless tied to operational definitions. They should be validated against longitudinal outcomes—innovation uptake, retention of knowledge—and aligned with existing findings that partially connected networks help preserve diversity (Derex and Boyd, 2016), thereby providing a coherent evaluative framework. Third, while the “Goldilocks” hypothesis for creative engagement is promising, the optimal uncertainty is likely task-, expertise-, and risk-dependent, plausibly nonlinear and hysteretic. Infant learning and musical groove studies establish the principle (Kidd et al., 2012; Vuust et al., 2022b), but parameterizing it for complex adult decisions will require closed-loop, adaptively tuned experimental designs. Fourth, our AI proposals—synchrony-like interaction signals and bio-informed sensory–affective alignment—are, at best, proxies for interoception rather than true interoceptive generative models. Their limits must be quantified against strong non-embodied baselines to demonstrate the incremental value of embodied proxies. Finally, there is no standard benchmark for FLI. Community-agreed tasks and metrics are needed to jointly test collapse resistance under self-generated data, diversity preservation, and socially accepted novelty under partial connectivity; without such shared benchmarks, cumulative progress will be difficult.

Acknowledging these constraints, this paper nevertheless advances a framework that centers FLI—beyond individual internal models—as a key to the persistence of creativity in human societies. Using music as a tractable model, this paper has highlighted how dynamic fluctuations between prediction and deviation are socially shared via bodily sensation and empathy, and how this circulation sustains creative capacity. This paper further argued that these insights can be extended to scientific collaboration, education, and human–AI co-creativity, offering a theoretical basis for explaining why creativity does not merely endure across generations but is actively renewed.

## 5 Conclusion

Creativity persists in human societies not as a property of isolated minds but as a phenomenon distributed across bodies, artifacts, and social relations. Viewing creativity through the lens of predictive processing and musical practice reveals a simple but powerful logic: moderate, time-varying uncertainty ignites exploration; interoceptive sensation and empathic uptake translate deviation into shared value; and partially connected social structures preserve diversity while preventing premature convergence. In this dynamic, music functions as a collective prediction device, making visible a putative operational signature of FLI.

This review synthesized evidence that, in music and related coordination-rich settings, prediction–deviation dynamics are stabilized and renewed by bodily synchrony, shared grammars, and socially negotiated admissibility. It further proposes—rather than demonstrates—that some of these mechanisms may generalize as design principles, provided that domain-specific norms, risks, and ethical costs are explicitly modeled.

A concrete research agenda follows. First, formalize field-level constructs—social precision weighting, admissibility of deviation, collapse resistance—into operational metrics and community benchmarks. Second, develop closed-loop, ecologically valid experiments that manipulate uncertainty and synchrony while tracking longitudinal outcomes such as innovation uptake and knowledge retention. Third, prototype sensory–affective alignment layers that are strictly opt-in, privacy-preserving, and auditable, and evaluate their value over strong non-embodied baselines. Finally, embed these components within collective predictive coding frameworks so that meanings and norms can emerge and stabilize through interaction rather than prescription.

In sum, the persistence of creativity is supported not solely within individual brains but across the field—through interoceptive, empathic coupling and culturally shared musical grammars and bodily maps. Music makes these dynamics especially visible: moderate uncertainty elicits a sense of creativity that, via bodily synchrony and empathy, is socially shared and stabilized. Within music, this may represent an operational signature of FLI; whether comparable signatures hold in other creative domains remains an empirical question.

## Data Availability

The original contributions presented in the study are included in the article/Supplementary material, further inquiries can be directed to the corresponding author.
