# Supplementary material for: Towards a theory of human creativity sustained by embodied collective intelligence
Source: Front Psychol. 2026 Apr 9;17:1752280. doi: 10.3389/fpsyg.2026.1752280 (PMC13102783; doi:10.3389/fpsyg.2026.1752280)
Supplement: Supplementary file 1 [file Supplementary_file_1.docx]

**Appendix A. Glossary of Key Constructs**

This glossary provides concise working definitions of key constructs used in this review.

- **Uncertainty:** Unpredictability of upcoming events under a predictive model, often quantified as entropy of the predictive distribution.
- **Surprise:** Violation of expectations, commonly formalized as prediction error or information content (e.g., −log probability of an event under the model).
- **Precision (weighting):** Confidence assigned to prediction errors (roughly the inverse of uncertainty) that determines how strongly errors influence updating and action.
- **Interoception:** Perception and awareness of internal bodily states (e.g., cardiac, respiratory, visceral sensations).
- **Interoceptive inference:** Predictive process through which the brain estimates and regulates internal bodily states by minimizing interoceptive prediction errors, shaping emotion and self-related experience.
- **Bodily synchrony / physiological coupling:** Temporal alignment between individuals’ movements and/or physiological signals (e.g., heart rate, respiration) during interaction.
- **Empathy:** Processes enabling understanding and sharing of others’ states; used here to include both affective sharing and cognitive perspective taking.
- **Perspective taking:** Cognitive capacity to model another person’s viewpoint, intentions, and feelings, supporting mutual understanding and coordination.
- **Admissibility of deviation:** A field-level acceptance boundary that determines whether a novel deviation from shared expectations/norms is admitted, reshaped, or rejected by the group.
- **Field-level intelligence:** Emergent collective cognition arising from interaction (rather than simple aggregation) that preserves diversity and maintains ‘just-right’ uncertainty over time.
- **Social precision weighting:** Collective modulation of how much weight (precision) is assigned to a deviation, often mediated by empathic uptake and normative evaluation, shaping whether novelty becomes socially shareable.

**Table 1. Key constructs, operational definitions, and example falsifiable predictions.**

| **Construct** | **Working definition** | **Candidate operationalization / proxies** |
| --- | --- | --- |
| Bodily synchrony / physiological coupling | Temporal alignment between individuals’ movements and/or physiological signals during interaction. | Phase-locking/cross-correlation of movement; HR/respiration synchrony; inter-subject neural coupling. |
| Interoceptive inference | Predictive process that estimates and regulates internal bodily states by minimizing interoceptive prediction errors. | Heartbeat-evoked potentials; insula activity; interoceptive accuracy/sensibility; perturbation via haptic feedback. |
| Empathy / perspective taking | Affective sharing and cognitive modeling of others that shapes interpretation and acceptance of novelty. | IRI subscales; empathic accuracy tasks; prosocial choices; self–other overlap measures. |
| Admissibility of deviation | Field-level acceptance boundary for novelty (admitted/reshaped/rejected). | Acceptability/ethics ratings; sanction decisions; adoption/retention; network diffusion metrics. |
| Field-level intelligence | Emergent collective cognition that maintains diversity and ‘just-right’ uncertainty. | Group-level innovation uptake; diversity indices; robustness to collapse; partially connected network effects. |
| Just-right uncertainty | A regime in which the entropy and prediction-error variability are intermediate—neither depleted nor chaotic—supporting engagement and exploration. | Entropy rate of sequences; prediction-error variance; subjective interest/engagement; exploration/exploitation balance. |
